# Health optimisation for patients with obesity before elective orthopaedic surgery: a qualitative study of professionals’ views on restrictive approaches and future practice

**DOI:** 10.1186/s13741-024-00460-1

**Published:** 2024-10-18

**Authors:** Joanna McLaughlin, Ruth Kipping, Hugh McLeod, Andrew Judge, Amanda Owen-Smith

**Affiliations:** 1 Bristol Medical School, Translational Health Sciences, Musculoskeletal Research Unit, Southmead Hospital, University of Bristol, Learning and Research Building, Level 1, Bristol, BS10 5NB UK; 2 Bristol Medical School, Population Health Sciences, University of Bristol, Bristol, BS8 2PS UK; 3 National Institute for Health and Care Research Applied Research Collaboration West (NIHR ARC West), University Hospitals Bristol and Weston NHS Foundation Trust, Bristol, BS1 2NT UK; 4 National Institute for Health and Care Research Bristol Biomedical Research Centre, University Hospitals Bristol and Weston NHS Foundation Trust, University of Bristol, Bristol, UK; 5 Nuffield Department of Orthopaedics, Rheumatology and Musculoskeletal Sciences (NDORMS), University of Oxford, Oxford, UK

**Keywords:** Health optimisation, Obesity, Body mass index, Weight management, Elective surgery, Pre-operative, Policy, Qualitative, Arthroplasty

## Abstract

**Background:**

Preoperative health optimisation for elective surgery entails supporting patients to improve their health in preparation for their treatment and recovery. While there is consensus that this process should address obesity, approaches vary across England. Despite guidance from the National Institute for Health and Care Excellence to the contrary, restrictive approaches with body mass index thresholds for referral to arthroplasty are in use. This qualitative study aimed to investigate the views of professionals on the current use and future implications of these policies.

**Methods:**

Semi-structured interviews were conducted with 20 professionals including clinicians, commissioners, policymakers, and health service managers, with experience of developing and/or implementing health optimisation policies for elective arthroplasty. Participants were sampled from areas in England with and without restrictive policies. We undertook thematic analysis of the interview data.

**Results:**

Participants described pre-surgical health optimisation as an important trigger for health improvement but identified current resourcing and inadequacies in provision of weight management support as significant barriers to success. Participants expressed concerns about the appropriateness and fairness of including obesity as a determinant to restrict access to surgery. They described short-term financial pressures underlying the use of restrictive body mass index thresholds and a lack of an evidence base, such that policies amounted to rationing and risked exacerbations of health inequalities. The study identified four priorities for improvements to future health optimisation practices: developing and implementing national guidance with flexibility for local variation, initiating patient engagement in primary care with onward integration across all services, improving resourcing to support effective equitable impact, and addressing wider determinants of obesity through societal change.

**Conclusions:**

Overall, participants had limited expectations of the impact of health optimisation policies on obesity without additional support, investment, and national guideline implementation. They raised strong concerns over current restrictive approaches. We conclude that addressing concerns around weight management support service availability and impacts on health inequalities is essential for shaping effective health optimisation policies. Future policy direction should support health optimisation to be offered early (ideally in primary care). Health optimisation interventions should be non-restrictive, inclusive, and well-monitored, particularly around equality impact.

**Supplementary Information:**

The online version contains supplementary material available at 10.1186/s13741-024-00460-1.

## Background

Perioperative health optimisation prior to elective surgery is a policy priority in England’s National Health Service (NHS) as well as in international settings (NHS England 2023a; Patel et al. 2022). NHS guidance defines health optimisation as ‘the process of supporting and working with a patient to improve their health before surgery. This includes both supporting people with the management of any long-term conditions and supporting people with any behaviour changes required to improve their health’ (NHS England 2023a).

Leveraging the ‘teachable moment’ for a person contemplating surgery may support them to address modifiable risk factors such as obesity through behaviour change and/or engagement with weight management services (Howard et al. 2023). Potential benefits from reducing obesity include lower need for surgery, improvements in surgical outcomes and in longer-term health and wellbeing measures (Durrand et al. 2019). Policymakers may choose to use health optimisation policies which introduce body mass index (BMI) thresholds to alter patient pathways to surgery (McLaughlin et al. 2023).

In England, the power to create and apply such policies lies with the 42 locality-based commissioning groups known as integrated care boards (ICBs). The autonomy afforded to ICBs means that their policies diverge by geography and are not always aligned with national guidance (Rooshenas et al. 2022). Due to the high numbers of patients needing elective arthroplasty each year (circa 100,000 hip and 100,000 knee replacements were undertaken in the UK in 2022 (Achakri et al. 2023)) and concerns over the costs of obesity-related surgical complications and post-operative care (Nightingale et al. 2015), guidelines and policies regarding obesity are prominent in this setting (NHS England 2023a; Durrand et al. 2019; McNally et al. 2021). Obesity is common in this group of patients (41% of hip and 56% of knee replacement patients had a BMI ≥ 30 kg/m^2^ in 2022 (Achakri et al. 2023)), meaning BMI threshold policies have an impact on many thousands of patients. There has been a 64% increase in the number of patients on waiting lists for trauma and orthopaedics referrals in England between 2020 and 2023 (Warner and Zaranko 2024).

The United Kingdom’s National Institute for Health and Care Excellence (NICE) is a public body which provides national clinical guidance (National Institute for Health and Care Excellence 2023). It is intended to provide a rigorous evidence-based process for guideline creation, removing some of the burdens on local settings to synthesise evidence and to negotiate interpretation and decision-making.

NICE guidelines for osteoarthritis management are clear that due to concerns over health inequalities and a lack of clinical justification, BMI should not preclude patients from referral to arthroplasty (National Institute for Health and Care Excellence 2022). Despite this, around half of commissioning localities in England in 2021 had a restrictive policy regarding BMI in place for arthroplasty, typically a BMI threshold for eligibility for surgical referral (McLaughlin et al. 2023). Other localities had either no mention of obesity or BMI in their arthroplasty policy or used nonrestrictive policies simply offering weight management advice and support. However, the supporting evidence behind these decisions remains unclear.

Concerns over the use of restrictive policies have been noted in media coverage, academic ethics publications, and expert commentary since 2016 (Pillutla et al. 2018; Royal College of Surgeons 2016). The use of BMI thresholds has been characterised as an unjust form of rationing, targeting patients living with obesity who already face stigma and health inequalities. Further concerns centre on the limited coverage, capacity, and efficacy of the weight management services offered to patients whose surgical referral has been delayed or denied through the implementation of restrictive health optimisation policies (Local Government Association 2018; Office for Health Improvement and Disparities 2022).

In this study, we aimed to investigate these concerns by talking to professionals to understand how and why restrictive policies were developed and implemented and gather insights about priorities for future policy development in this area.

## Methods

This study focused on perioperative optimisation policies for patients with obesity considering elective arthroplasty. We used semi-structured interviews to investigate the views of clinical and health managerial participants. The research was undertaken within an overarching paradigm of interpretivism, seeking to explore the meanings and beliefs that individuals ascribe to their experiences (Ritchie et al. 2013). The study protocol was not registered. The study received approval from an NHS Research Ethics Committee, and details are in the “Declarations” section.

### Setting and participants

Potential participants were selected through purposive sampling, which is a targeted approach seeking to recruit participants with a range of perspectives relevant to the phenomena under study (King et al. 2019). The sampling frame included commissioners, policymakers, managers, and clinicians from different areas of England with and without restrictive health optimisation policies in place. Other individuals working within national organisations (NHS England and the Centre for Perioperative Care) with a remit in perioperative optimisation and prehabilitation policy development and implementation were also approached to participate. Further invitations were made where participants suggested other potential participants (snowballing (Ritchie et al. 2013)) with relevant involvement in health optimisation in divergent policy regions of England.

Invitations were issued by email, and potential participants provided responses directly to the main researcher. Data collection and eventual sample size were informed by the concept of ‘information power’ (Malterud et al. 2016) with sampling, participant recruitment, and analysis conducted in parallel to allow a continuous assessment of the data collected. When the concurrent data analysis suggested a consolidation of emergent themes and multiple participants from each professional group had been interviewed, sampling was deemed to be complete. This approach to determining data adequacy (rather than anticipating ‘data saturation’) was necessary as the interview guide contained a broad scope and the potential pool of participants was very diverse.

### Interviews

Semi-structured interviews were conducted using a topic guide informed by relevant literature, the authors’ prior professional experience, and patient and public involvement. The topic guide is detailed in Additional file 1. It was not pilot-tested. The main topics were as follows: the current evidence base for health optimisation, concerns around inequalities in implementation, best practice in health optimisation, and future development needs. Interviews were conducted by J.M. via video call (MS Teams) or in person at the participants’ preference. No nonparticipants were present. All were audio-recorded on an encrypted digital audio recorder and then fully transcribed and anonymised by the interviewer.

### Data analysis

All transcripts were read and reread to gain familiarity with the data, and thematic analysis of the data was undertaken based on recommendations described by Braun and Clarke (2006). Initial ideas were documented before open coding using qualitative data analysis software (NVivo) was applied to blocks of text by J.M. Independent coding of a subsample (*n* = 5) of transcripts by A.O-S. was undertaken to enhance the rigour of analysis; differences in interpretation were discussed until agreement was reached for an initial coding framework which was then applied to all transcripts. J.M. has a clinical background, and the potential impact of this was discussed by the research team to provide reflexivity in the interpretation of the data analysis. In addition, A.O-S. (qualitative lead) has completed numerous previous studies related to rationing and access to treatments for obesity, which inevitably influenced her perspective around appropriate frameworks for the allocation of scarce resources. The research team had previously encountered seven of the interviewees in the course of their professional careers.

Data analysis ran in parallel with sampling and data collection so that emerging themes could be followed up and synthesised. The coding framework was kept under review and updated to this effect, with re-coding of earlier transcripts where necessary. The coding framework is provided in Additional file 2. Negative cases, where participants held divergent views or experiences, were re-analysed to gain further insights. Codes were amalgamated into major themes, for example, a theme of ‘drivers for policy introduction’ was formed from the data coded regarding policy use for ‘patient benefit’, ‘financial or rationing’, and ‘acceptability and ease of policy introduction’. Matrices were then created to show the coded extracts by source for each theme. Further analysis of these matrices showed how the themes related to each other and allowed the comparison of findings across participant groups and between individual participants.

### Public and patient involvement

The study concept, topic guide, and recruitment materials were developed with the involvement of a patient and public group with lived experience of arthritis and joint replacement. Discussion of the main findings of the data analysis with the group informed the reporting of the results and their policy, practice, and further research implications.

## Results

### Participants

Twenty of 25 invitees agreed to participate and completed an interview. One participant opted for an in-person interview at their workplace; the remainder was conducted via video call. The interviews lasted for a median of 45 min (range 37 to 56 min). Interviews took place between June and September 2022.

Participants worked across seven areas of England, but some also drew on experiences of recent employment in other areas, and thus, participants had insights into restrictive and nonrestrictive policies. The participants’ primary roles (those in which they spent the majority of their professional time) are shown in Table 1. Many participants identified themselves as holding a number of roles. For example, the general practitioners had past or present commissioning responsibilities, and seven of the eight commissioners and policymakers had clinical backgrounds and experience.

**Table 1:** Study participants’ primary roles

Primary role	*n*
General practitioners	3
Orthopaedic surgeons	3
Public health professionals	2
Other secondary care clinicians (anaesthetists, geriatricians)	4
Commissioners and policymakers	8

### Analysis findings

Three primary themes are presented from the analysis: Firstly, insights into the development and implementation of restrictive policies; secondly, concerns over the impact of restrictive approaches; and thirdly, the priorities for change in future policy and practice for health optimisation for obesity.

Data are presented with the use of illustrative quotes labelled with the participants’ number and ‘managerial’, ‘clinical’, or ‘both’ to denote whether their current role was managerial, clinical, or both respectively. Ellipses […] are used to denote text omitted for the purposes of clarity and brevity.

#### The development and implementation of restrictive policies

There was broad agreement between participants that the concept of addressing obesity in the surgical pathway was legitimate and important, with 14 participants directly stating that weight loss could improve surgery safety and short-term outcomes.

You are likely to recover better from surgery if you were a healthier weight. (I14 - Managerial)

In two other accounts, managerial participants spoke of an intent to reduce the need for surgery whereby health optimisation policies trigger improvements in arthritis symptoms due to weight loss.

If you help them lose the weight, for example, someone who's got knee pain doesn't necessarily have knee pain anymore. (I7 - Managerial)

#### Financial pressures

The primary driver for the use of *restrictive* policies was described as that of short-term financial pressures. All participants mentioned financial considerations within their accounts, though there was variation in the support expressed for this rationale and in their confidence that financial benefits would actually accrue. Many participants described the use of restrictive policies as tools in the localised rationing of elective surgery in response to NHS resources and waiting list pressures.

If you can only provide limited resource across every service, you do need to prioritise and decide if you’re going to give a knee operation to everyone, or whether you're gonna have some cuts off and pick the winners. (I10 - Clinical)

It's a sort of un-thought-through fairly blunt, financially oriented tool for saving money […] some bright spark in a management consultancy type of role will have sort of done some back of fag packet calculations. […] work out how many people are on the elective waiting list who are overweight. (I8 - Managerial)

One clinician identified an explicit consideration of encouraging or requiring patients to pay for their own treatment if their BMI fell into an overweight category, which exposed the potential for health optimisation policies acting as rationing tools.

If you want to qualify for your hip or knee replacement, you just can't have it done free unless your BMI is 30. If you wanna have it done at BMI 35 go and see a private surgeon. (I17 - Clinical)

The strength of these financial and rationing motivations was described by several participants as exacerbated in recent times by pressures on the NHS and the wider economy.

They’re a thing that we've seen grow over time in terms of their use, particularly as financial pressures on the NHS have increased […] NHS finance directors are just going, no, I don't care. […] everything's on fire and I just have to try and make the books balance. (I14 - Managerial)

Participants described political and public acceptability of rationing based on obesity in relation to societal framing of obesity as a personal responsibility. Three participants supported the legitimacy of patients’ accountability for weight management, while most others considered this ‘blame’ basis to be a flawed justification for rationing, used nonetheless by commissioners facing severe financial pressures.

There's an element of personal accountability [for obesity…] a lot of patients don't want to take responsibility for their health. They see it as their personal right to make lifestyle choices, which clearly it is. But then they want to have the benefits of the health care as well if something goes wrong. (I17 - Clinical)

[Commissioners are] doing it because they're rationing where they think they can ration care, and they get away with it because they pick services where blame can be attributed. (I14 – Clinical)

#### The evidence base

The evidence base for health optimisation approaches overall was another theme prominent in participants’ accounts of the reasons behind differing policy choices. The evidence base was described as immature and lacking in local specificity, with few published evaluations of health optimisation programmes for commissioners to work from. Participants interpreted the regional variation in policy use as a reflection of this limited evidence-base.

Where there’s big variation in anything, that generally suggests that there isn't a clear right or wrong way of doing things. Or if there is, people aren't aware of it […] Therefore organisations and areas create their own approaches. (I3 - Both)

As such, decision-makers’ own interpretations of the evidence and willingness to introduce policies of unproven value were felt to have influenced the choice of policy type in different areas.

Particularly with an area like BMI where there is a lot of, um, controversy […] I think it's very much kind of down to the group who are interpreting that evidence - their experiences, their sort of personal experience and prejudices. (I6 - Clinical)

It becomes more of a political conversation […] if people need to make decisions […] I've seen examples of where people sort of fit scientific evidence and or lack of it, to managerial [needs]. (I8 - Managerial)

#### Participants’ concerns over the impact of restrictive policies

Core concerns related to restrictive policy use were as follows: the lack of BMI-based justification for delaying surgery, harm to the clinical autonomy required for effective shared decision-making, inadequacies in the current health service infrastructure to support lasting weight loss, and exacerbations of health inequalities.

#### Concerns about the validity of BMI as a clinical indicator

Clinical participants were not wholly positive about the validity of using surgical outcomes as a rationale for weight loss through health optimisation, and concerns were expressed that delays to surgery could result in worsening health.

If you end up delaying their surgery to try and get them down to that BMI then their pathology progresses, […] the longer people are in pain […] the poorer their pain outcomes are after surgery […] there’s potentially some real pitfalls. (I6 - Clinical)

Exceptions were made in very high ranges of BMI where immediate anaesthetic risk and surgical practicalities became significant issues, but overall, there was low support offered for the clinical validity of hard cut-offs in BMI by participants from all groups.

When they’re really, really big, risks then do go up significantly […] I believe there is an upper limit whereby it is probably not wise to proceed. (I11 - Clinical)

What's the difference between a BMI of 34.9 and a BMI of 35.1? Probably not very significant, but yet we've picked an arbitrary cut-off.” (I10 - Clinical)

Two participants also noted concerns of malnutrition or psychological health where they perceived that patients felt they must engage in crash diets to achieve surgical referral.

You don't want people to just restrict calories down and down and down to lose weight because they can lose muscle mass as well. (I6 - Clinical)

#### Concerns about communication with patients

A manager described the concern raised to them by frontline clinicians who opposed policies which placed them as gatekeepers. They worried that the need for the clinician to communicate the restrictive measures to patients would be damaging to the clinician-patient relationship.

If you turn us into the police, who determine whether you can have an operation or not based on your weight and exercise. You're going to give us a bad reputation with patients. (I1 - Managerial)

Despite this concern, several participants described that restrictive or even punitive approaches were potentially an effective tool in interactions with patients to increase engagement with health improvement for the patient’s own benefit.

We want to reduce obesity and we want to take opportunities to do that and patients waiting for surgery, it might be seen as a lever and so you know, that teachable moment in a slightly more punitive way, as it were, gives patients something to aim at. (I12 - Both)

A need to retain clinical autonomy, and to share meaningful communication on the risks of higher BMIs in individual patient assessments, was the preferred approach to ensure appropriate shared decision-making.

I'm not really not convinced in the sort of moral and scientific validity in applying that sort of anaesthetic risk in a sort of a ‘what should the policy on this be for a cohort of people?’ It's a sort of a patient by patient, person, type of conversation that needs to be part of an informed consent process. (I8 - Managerial)

#### Concerns about the wider healthcare infrastructure’s ability to support weight loss

Participants expressed scepticism over the effectiveness of health optimisation in practice in the current climate of very long waiting lists and the inability to give patients a surgical date to work towards.

If people don't see an endpoint to their timing for surgery, they disengage from what you're asking them to do because they don't see the point. (I17 - Clinical)

In addition, suitably well-resourced support services for behavioural change were felt to be lacking or inappropriate for all patients.

I think, lots of people are helped by things like Weight Watchers and all the rest of it. But if you are a single parent with three kids, you can’t get to Weight Watchers […] blanket rules don’t work. You have to tailor your interventions to the patients that you’re treating. (I12 - Both)

The obesogenic environment was cited as another reason for low confidence in significant and lasting weight loss achieved through health service engagement, particularly for people in deprived socioeconomic circumstances.

About 10% of them [attending community weight management services] lose 5% body weight, which is not insignificant. A lot of them then put it back on because they live in obesogenic environments. (I8 - Managerial)

This is not people who aren't trying to lose weight but as I say they are, they are not able to do that via knowledge or just the economic situation they find themselves and they're not empowered to make the change.” (I16 - Managerial)

#### Concerns about exacerbating health inequalities

Health inequalities were a key concern for many participants. In particular, participants raised concerns that geographical inequalities would result due to some regions having more restrictive policies or poorer weight management support services than others.

I don't agree with it for lots of reasons. […] it's a blunt tool, […] I think it will amplify health inequalities. […] I guess whoever wrote those policies must have assumed that patients would be given help to reduce their weight […] but I doubt very much that that's consistent. (I12 - Both)

By applying the ‘you can't have your hip operation until you've lost weight you fatso’ […] less poor people are going to get hip operations and that's not right. Dear The NHS, you have a legal duty to redress inequalities. This policy is structurally failing that duty.” (I8 - Managerial)

The ability for some to fund their own private treatment and avoid delay to their surgery should they wish was also raised as a driver of health inequalities that would result from restrictive policy use.

People on waiting lists, 65% say that private care is simply not an option for them […] it will be the people who are in poverty, who are from ethnic minorities, women, who will be affected the most by that. And so this two-tier system of more well-off people gonna end up going private. (I14 - Managerial)

#### Future priorities

Themes in participants’ accounts were grouped as four key elements regarding progress towards best practice for obesity health optimisation in the NHS.

Table 2 illustrates these elements and the specific recommendations to meet them, with sample quotations from the analysis.

**Table 2:** Summary of key elements and recommendations identified by participants for best practice in health optimisation in the future (M managerial, C clinical, B both)

Key element	Recommendations	Example quotes
**1. National guidance with room for local variation**	Create national, obesity-specific health optimisation guidance with local flexibility	“If there was a single policy, then it'd be more likely to be acted upon and resourced […] It would cut down on perceived unfairness.” (I3—B) “Flexibility is crucial, […]That is the aim of having localised healthcare systems.” (I5—M)
	Cease restriction by BMI thresholds, to reflect NICE guidance	“There isn't a [BMI] cut off, when you look at outcomes, it's a continuum […] for a lot of these patients, you’re just denying them the care” (I11—C)
	Address evidence gaps in health inequalities and long-term impact	“So they're off the waiting list. Fantastic […] let's not stop there, let's continue to monitor a cohort of individuals who are undergoing some of these interventions and see what the long-term implications are.” (I16—M)
**2. Position in healthcare: health optimisation as everyone’s business**	Offer earlier, integrated health improvement, initiated in primary care	“We're [GPs] well situated to have those conversations about needing to be as fit as possible for an operation” (I6—C) “Social prescribers, GPs, community providers, third sector providers, local county councils, […] personalised care. […] it, should be part of everybody's programme.” (I7—M)
	Include health optimisation onto clinical education curricula	“Perioperative medicine isn't really taught in medical school […] surgeons can be very focused on their one specialty (I9—M)
	Reduce the current emphasis on surgical patients	“We want to do more of this pre and post-op and way outside the context of elective surgery as well.” (I8—M)
**3. Better resourcing and data system developments**	Invest in effective, accessible support services that cater to individual need	“If the NHS wants to increase the throughput of […community] weight management services, that's great […], but I haven't got any more money to put into it.” (I8—M) “[we need] a massive increase in funding and availability of support services” (I13 -C)
	Modernise digital infrastructure to allow integrated care and evaluation	“We know what we're seeing at secondary care level fairly well, but there isn't that same level of knowledge at primary care and the ability to tie that into health inequalities data.” (I16—M)
	Leverage integrated care systems for a more systemic approach to health optimisation	“We're seeing in the shift with our ICSs, […] how you use the total pot of money you've got, to manage for the best outcomes of your local population rather than how many hip replacements you've successfully delivered.” (I14—M)
**4. Societal change and wider determinants**	Address the impact of environment and opportunities before people reach the health service	“We need to be working with people at every stage of their lives […] around what people can do to improve their health, and what is and isn't possible through healthcare.” (I15—B) “[We need a] supportive approach […] one that addresses and takes the social determinants into consideration.” (I19—M)

Overall, a vision of health optimisation offered early in care pathways, integrated across all sectors, with an inclusive, nonrestrictive approach centred on shared decision-making, protecting against exacerbations of health inequalities, was apparent. Primary care was raised as the most suitable setting for the start of a patient’s engagement with health optimisation, though several managerial participants cautioned that this may be asking too much of an already highly pressurised service.

Participants argued that evidence-based national guidelines could play a role in improving health optimisation practice while leaving room for beneficial local variation in approaches, e.g. referral pathways tailored around existing local services. Many participants emphasised that progress would be reliant on adequate resourcing for implementation. There were particular concerns over the investment needed to improve access to suitable weight management support and to provide the data and digital systems necessary to make health optimisation pathways work efficiently. Accounts indicated that there was also an awareness that health optimisation can account for only one avenue for addressing obesity and health improvement more generally — societal change and action on the wider determinants of health must play their role.

## Discussion

### Summary of key findings

This study used interviews with key clinical and managerial participants to investigate the use of health optimisation policies which, contrary to national guidance, restrict patient access to arthroplasty surgery based on their body mass index.

A central finding was that restrictive policies were often considered as a form of rationing driven primarily by NHS financial pressures. Restrictive policies were viewed as justified by some commissioners on the grounds that pre-surgical obesity management benefits patient outcomes and obesity can be seen as an issue of personal responsibility. A lack of evidence over policy impact, and a perceived tendency for decision-makers to cherry-pick evidence to support their proposals, encouraged restrictive policy use. However, participants had strong concerns over the continued use of restrictive policies for a number of reasons. These included a lack of clinical justification for rationing by BMI, the inadequacies of available weight management support in an obesogenic environment, and the worsening of health inequalities. This last element related to the use of private surgery to bypass NHS restrictions by those patients who can afford it.

Four areas of recommendations for future practice and policy were identified: creation of national guidance, positioning health optimisation as everyone’s business, resourcing integrated support services, and addressing wider determinants of obesity.

### Strengths and limitations

The major strength of this qualitative study was the ability to capture views and insights from a broad range of participants in significant professional roles relating to the design and implementation of health optimisation strategies. Semi-structured interviews permitted in-depth exploration of a complex policy environment and detailed investigation of divergent views and experiences. Sampling across different geographical areas in the context of a variation-filled policy landscape allowed the analysis to bring together the major elements common to underlying attitudes and decision-making while exploring the differences in policy impact.

Limitations include the restriction of the study to publicly funded healthcare provision in England, which is a highly centralised service supported by an expanding private healthcare market. Views about the appropriateness and impact of health optimisation policies will likely vary within other healthcare system settings; however, many of the evidence gaps and concerns over equity identified in this study remain applicable. The interviews were completed in 2022, but with restrictive policies still in current use in England and 7.5 million NHS patients on waiting lists for elective surgery (Warner and Zaranko 2024; The King’s Fund 2024), the findings of this study are highly relevant to current patient experiences.

This study did not directly address patient and public views on health optimisation. Previous studies, including our research, have reported related qualitative research with patient participants, and public consultations have reported public opinion on health optimisation approaches demonstrating a dichotomy in opinions reflecting either the personal responsibility framing of obesity as a justification for restriction or concerns over unfair two-tier access to healthcare related to existing health inequalities (McLaughlin et al. 2021; Avery-Phipps et al. 2022).

### Comparison with existing literature

This study supplements existing literature detailing an ‘evidence-policy’ gap in healthcare commissioning, whereby multiple factors play into decision-making, including ‘the tendency of policymakers to base judgements on their beliefs, and shortcuts based on their emotions and familiarity with information’ (Cairney et al. 2017). Where policies are created in the setting of a localised organisational structure, there is a push–pull between evidence quality and relevance, and this is reflected in the experiences and interpretations of the participants in this study.

A recent analysis of policies for accessing elective musculoskeletal procedures in the NHS in England examined variation across 14 localities (Rooshenas et al. 2022). The study concluded that evidence was cited inconsistently with recurrent variation in specifications and requirements, despite the existence of NICE guidance. This is reflective of the current, localised policies which diverge from NICE guidance on BMI thresholds in arthroplasty described in this study. The authors suggested that more central support is required to promote consistency, and that this is critical where the evidence base is deemed uncertain for a particular policy (Rooshenas et al. 2022). Our study’s findings show participants’ appetite for additional national guidance to shape pre-surgical health optimisation approaches to obesity, informed by improvements to the evidence base for different approaches.

This study identified the impact of policy interventions on health inequalities as a key consideration in health optimisation’s future. When implemented in a nonrestrictive approach, participants were optimistic that health optimisation could reduce health inequalities. However, within restrictive approaches which frame obesity as a personal responsibility, exacerbations to health inequalities were described. The legitimacy of the personal responsibility argument, used by commissioners to justify restrictive policy use, is not supported by authors in reviews of the ethical dimensions of policymaking for restrictions in access to healthcare (Coggon 2021; Sharkey and Gillam 2010). The inadequacy of any individual-level approach to reduce obesity prevalence is well-documented—action on the obesogenic environment for whole communities is essential (McNally 2023).

Concerns over the use of BMI as a measure in itself, and as a tool for application of rationing, are already well-reported in the literature (Gutin 2021). Acknowledgement of BMI as an imperfect measure which is ‘available’ rather than ‘desirable’ reflects the views of participants in this study that BMI serves to create a practical, objective threshold, but one which lacks clinical credibility in decisions determining meaningful risk and therefore justifiable denial of access to surgery.

### Policy, practice, and research implications

Where policies such as restriction of access to surgery by BMI so evidently contradict national guidance and have concerning implications for health inequalities, the case is clear to increase efforts to dissuade commissioners from continuing their use. Where sufficient clinical and public health opposition has been raised, there are examples of success in persuading commissioners to retract restrictive policies (Dodd et al. 2015). This retraction now needs to be addressed nationally and internationally.

While there are no current plans for specific national guidance on health optimisation for obesity, national requirements and policy provisions on perioperative care are increasing in line with an NHS England programme of work and the foundation of the Centre for Perioperative Care (2023). Whichever way weight management is introduced or incentivised in clinical pathways, the support services on offer must be adequately resourced. Existing support service provision was highlighted as inadequate in this study, and improved resourcing and tailoring to patients’ needs should be a major focus of efforts to address obesity in elective surgical pathways.

Participants recommended the earlier introduction of health optimisation into patients’ interactions with the NHS, initiated in primary care. It must be acknowledged that the feasibility of integration of additional health improvement activity into primary care would need to be addressed as a priority given the intense resource pressures already present in this sector.

There are many other relevant policy directives such as those requiring action on health inequalities which will shape the future choices made by commissioners regarding their approach to health improvement in the preoperative setting (NHS England). NHS initiatives such as ‘My Planned Care’ and elements in the NHS Elective Recovery Plan reflect the move towards universal, digitally centred support for patients on long waiting lists which may also influence access to health improvement support for NHS patients (NHS England 2022, 2023b).

## Conclusion

This study highlights the problematic continued use of restrictive health optimisation policies which ration access to orthopaedic surgery on the basis of BMI. Recommendations drawn from participants’ experiences and insights promote the production of national or international guidance on weight management in pre-surgical care. This should support health optimisation to be early, non-restrictive, inclusive, and well monitored including for impact on inequalities. Significant investment is required in weight management support and related research if pre-surgical health optimisation policies are to fulfil their potential in improving population health through action on obesity.

## Supplementary Information

Additional file 1. Outline topic guide for semi-structured interviews.

Additional file 2. Coding framework.

## Data Availability

The datasets generated and/or analysed during the current study are available from the corresponding author on reasonable request.

## Abbreviations

BMI: Body mass index
NICE: National Institute for Health and Care Excellence
NHS: National Health Service
GP: General practitioner
ICS: Integrated care system
ICB: Integrated care board
NIHR: National Institute for Health and Care Research
