# Supplementary material for: Health optimisation for patients with obesity before elective orthopaedic surgery: a qualitative study of professionals’ views on restrictive approaches and future practice
Source: Perioper Med (Lond). 2024 Oct 18;13:104. doi: 10.1186/s13741-024-00460-1 (PMC11488359; doi:10.1186/s13741-024-00460-1)
Supplement: Supplementary file 1 — Additional file 1. Outline topic guide for semi-structured interviews. [file 13741_2024_460_MOESM1_ESM.docx]

Additional file 1: Outline topic guide for semi-structured interviews

**Outline topic guide:**

Researcher introduces research, takes consent, etc.

Introduction / background

- To start off can you tell me a bit about your professional role?
  - Probe – experience with health optimisation policy/interventions
- What do you think of when you think about a typical health optimisation policy?
  - Probe – setting, target groups, support offered, thresholds and rules

Perspective on the current evidence base for health optimisation interventions

- How do you think the policies were developed?
- Ideological vs evidence based?
- How evidence-based do you think the current health optimisation policies are?
  - (If indicates that evidence base is lacking) What are the key gaps in the evidence base that concern you?
  - How typical is this evidence-base situation when you consider other health care policies?
- What are the key drivers for health optimisation policies?
  - Why are they introduced?
  - What is important in their continued use?

Inequalities

- How much emphasis do you think is given to the consideration of inequalities in health optimisation policy making?
  - Inequalities improved if interventions are inclusive and tailored? Or inequalities worsened if access to surgery is more difficult for some?

Perspective on best practice in health optimisation

- What works well in health optimisation policies?
  - Who do you think is best placed to raise health optimisation/health improvement opportunities with patients?
  - Which patient groups or conditions may be best served by health optimisation?
- What are the challenges with the policies?
  - Which patient groups or conditions may be most challenging for health optimisation?
  - Is health optimisation inappropriate in some settings and groups?

Perspective on current and historic health optimisation policy landscape

- Do you know of any differences between regions/anywhere that does it differently?
  - What do you think drives variation in health optimisation policies across the UK?
  - What is the consequence?
  - Awareness of media attention on the policies?
- Parallels with any other health care policy situation?

Perspective on the future of health optimisation and future research

- How widespread should health optimisation be in the NHS?
- How important is it for there to be consistency/a single NHS policy for health optimisation?
- (If indicates that there is a role for health optimisation policy in the NHS) What barriers to policy making need to be addressed?
  - Barriers to policy implementation and possible solutions
- What are the next steps in improving evidence-based policy making for health optimisation?
  - Further research that is needed
  - How best to communicate/disseminate evidence

Final questions and closure

*Researcher asks for any further comment, thanks participant and stops recorder*
