# Supplementary material for: Health optimisation for patients with obesity before elective orthopaedic surgery: a qualitative study of professionals’ views on restrictive approaches and future practice
Source: Perioper Med (Lond). 2024 Oct 18;13:104. doi: 10.1186/s13741-024-00460-1 (PMC11488359; doi:10.1186/s13741-024-00460-1)
Supplement: Supplementary file 2 — Additional file 2. Coding framework. [file 13741_2024_460_MOESM2_ESM.pdf]

Code	Subcodes	Detailed content and subcodes
1.0 Definition of health optimisation (HO)		broad, narrow or finite definitions offered by participants
2.0 Commissioning of HO		
2.1 barriers to policy intro	2.1.1 systemic issues	data or digital issues, lack of support service capacity, source of funding or ownership for HO efforts, impact of system payment by episode of care in secondary care
	2.1.2 opposition to HO policies	clinician or public health (PH) opposition, role of surgeon individual outcomes, legal contest or patient advocacy, media attention
	2.1.3 lack of quick wins	commissioning cycles, long term benefits unmeasured
2.2 drivers for policy introduction	2.2.1 financial or rationing	need for immediate impact on waiting lists, healthcare demand, reluctance over new outlays, power of finance directors
	2.2.2 acceptability and ease	ease and low cost of policy implementation, personal responsibility narrative, spread of policies between CCGs
	2.2.3 hoped for benefits to patients	health improvement (longer term or non-surgical, short term surgical or reduction in demand), systematise health improvement, impact on inequalities, patient experience
2.3 ethics justice and morality		rationing, discrimination, patient prioritisation, deservedness
2.4 impact of organisations	2.4.1 impact of ICS or ICBs changing NHS attitude	move from CCG landscape to integrated systems, approach to unification of policies within new organisations
	2.4.2 national requirements or policy	directives from NHSE or other national bodies
2.5 need for HO or prehab specialists		involvement or employment of HO or prehab specific staff

Code	Subcodes	Detailed content and subcodes
2.6 policy retraction	2.6.1 general	experience of retraction of any policies in NHS
	2.6.2 retraction of BMI threshold policies	specific experience of retraction in BMI threshold policies
2.7 reasons for variation	2.7.1 appetite for risk	
	2.7.2 spare funding	
	2.7.3 system structure allows it	tolerance or encouragement of regional variation
	2.7.4 differences in demographics	
	2.7.5 engagement with public health	
	2.7.6 enthusiasts and advocates	and in contrast, vocal dissenters
	2.7.7 evidence isn't clear	
	2.7.8 political will and organisational priorities	
	2.7.9 service and financial pressures	pre-existing differences in resource, support service or related service capacity and demand, existing surgical demand and waiting lists, role of special measures
2.8 role of evidence base	2.8.1 BMI	BMI as a practical or managerial consideration, BMI as a risk factor or BMI reduction as a benefit, benefits of exercise, need for holistic approach, problematic weight loss malnourishment, use of BMI as a measure of obesity, use of BMI thresholds
	2.8.2 difficulty in HO evaluation	use of rate as outcome measure
	2.8.3 evidence is in support	confidence in evidence base for HO

Code	Subcodes	Detailed content and subcodes
	2.8.4 evidence is not in support or is lacking	concerns over evidence gaps, experience of policy introduction without evidence available
	2.8.5 need to be pragmatic and evidence informed rather than based	need for change without full evidence
	2.8.6 not up to date with the evidence	participants own assessment of their lack of knowledge base and that of policy makers and commissioners
	2.8.7 selective or inappropriate use of evidence	finding or shaping evidence to support pre-determined commissioning plans
3.0 Health optimisation delivery at present		
3.1 approach and practicalities	3.1.1 eligibility and timing for HO	patient identification, pathways, long term follow up
	3.1.2 employment of HO staff	use of specialist or existing staff
	3.1.3 issues with weight management and exercise interventions	difficulty knowing what exists, examples of successful and unsuccessful patients, support availability, efficacy of support, peer support or group settings, medicalisation of obesity and exercise, surgery needed for weight loss or exercising or difficulty exercising
	3.1.4 presentation to or reception by patients	clinician approaches (advice given, risk stratification and communication, setting a goal, shared decision making, patient choice, targeted approaches)<br><br>communication skills and trust (clinician prejudice & negative experiences of healthcare, patient initiated, patient perceived injustice or rationing)

Code	Subcodes	Detailed content and subcodes
		teachable moment, empowerment, selling the benefits (avoidance of surgery as a goal)<br>unwilling or uninterested patients (clinicians short on time)
3.2 COVID		impact on commissioning and evidence, impact on healthcare, move to digital or remote, negative impact of long waiting lists
3.3 Other related program and policy areas		wider or related perioperative, preventative, health improvement, waiting list management programmes
3.4 unofficial HO approaches		clinician or departmental approaches to HO in absence or contradiction of HO policy
3.5 Variation in delivery across UK		range and examples of HO programmes
4.0 Perceived impact of HO delivery		
4.1 health improvement		weight loss, smoking cessation, general health, mental health spill over impact to others
4.2 impact on clinician patient relationship		concerns over damage to relationship through difficult topics, unwelcome news, gatekeeping services
4.3 impact on healthcare service use	4.3.1 delay or reduced need for surgery	lengthening pathway to delay surgery, true reduction in need for surgery through symptom improvement
	4.3.2 readiness or fitness for surgery	importance of timing, importance of pre-surgical window, waiting well
	4.3.3 enforcement of rules	ability to circumvent policy, influx of out of area patients
	4.3.4 private surgery	patient motivation to pursue faster/accessible treatment, private

Code	Subcodes	Detailed content and subcodes
		healthcare providers rules on BMI and smoking
	4.3.5 inappropriate barrier to access	patients in need of treatment delayed, denied or poorer outcomes
4.4 Inequalities	4.4.1 HO negative impact on inequalities	postcode lottery of policy, rules and support<br><br>variation in engagement or accessibility (digital exclusion, socioeconomic deprivation, cost as a factor in engagement, cost of living worsening situation, impact on outcomes)
	4.4.2 HO positive impact on inequalities	tackling obesity and smoking disproportionately improves health in lower socioeconomic groups
5.0 Future direction and recommendations		
5.1 how HO should be delivered	5.1.1 where HO should be set	prevention and role of public health in HO, targeted or individualised approach, voluntary sector role, who is best placed to deliver HO
	5.1.2 making HO the default	Consideration of HO for all patients, all sectors
	5.1.3 mandatory nature of HO or offering choice	views on mandatory BMI policies, mandatory engagement with support, importance of patient choice
	5.1.4 new approaches - digital	wider reach with digital approaches, digital literacy needs
5.2 need for societal or broader change		active transport, obesogenic environment, intervention in childhood, wider determinants
5.3 what changes are needed to deliver HO well	5.3.1 need for support services and resource allocation	weight management and smoking cessation support service provision, financial support, recurrent funding, workforce

Code	Subcodes	Detailed content and subcodes
	5.3.2 need for national policy and evidence	value of national approach, guidelines vs. requirements, need for local flexibility
	5.3.3 need for synergistic synchronised alignment in message and support	approach across different sectors of health and care and patient encounters with multiple professionals and sectors
	5.3.4 medical education and staff health	HO in curricula, HO for staff, targeted training for HO delivery
	5.3.5 acceptability and advocacy	professional support for HO concept, communication of value
5.4 what HO should deliver		broader remit (everyone should be involved with HO, include post op and long term, not just smoking and weight)
